# Prognostic Role of Tumoral PD-L1 and IDO1 Expression, and Intratumoral CD8+ and FoxP3+ Lymphocyte Infiltrates in 132 Primary Cutaneous Merkel Cell Carcinomas

**DOI:** 10.3390/ijms22115489

**Published:** 2021-05-23

**Authors:** Piotr Donizy, Cheng-Lin Wu, Janusz Kopczynski, Malgorzata Pieniazek, Przemyslaw Biecek, Janusz Ryś, Mai P. Hoang

**Affiliations:** 1 Department of Clinical and Experimental Pathology, Wroclaw Medical University, 50-556 Wroclaw, Poland; piotrdonizy@wp.pl; 2 Department of Pathology, National Cheng Kung University Hospital, College of Medicine, National Cheng Kung University, Tainan 70403, Taiwan; wujl.towalkwithwings@gmail.com; 3 Department of Surgical Pathology, Holy Cross Cancer Centre, 25-734 Kielce, Poland; janusz.kopczynski@onkol.kielce.pl; 4 Department of Oncology and Division of Surgical Oncology, Wroclaw Medical University, 53-413 Wroclaw, Poland; malgorzatapienazek@interia.pl; 5 Department of Mathematics and Information Science, Warsaw University of Technology, 00-628 Warsaw, Poland; przemyslaw.biecek@gmail.com; 6 Department of Pathology, Center of Oncology, M. Sklodowska-Curie Memorial Institute, 31-115 Krakow, Poland; z5rys@cyf-kr.edu.pl; 7 Department of Pathology, Massachusetts General Hospital and Harvard Medical School, Boston, MA 02114, USA

**Keywords:** Merkel cell carcinoma, PD-L1, IDO1, CD8, FoxP3

## Abstract

The association of immune markers and clinicopathologic features and patient outcome has not been extensively studied in Merkel cell carcinoma (MCC). We correlated tumoral PD-L1 and IDO1 expression, and intratumoral CD8+ and FoxP3+ lymphocytes count with clinicopathologic variables, Merkel cell polyomavirus (MCPyV) status, and patient outcomes in a series of 132 MCC. By univariate analyses, tumoral PD-L1 expression >1% and combined tumoral PD-L1 >1% and high intratumoral FoxP3+ lymphocyte count correlated with improved overall survival (OS) (*p* = 0.016, 0.0072), MCC-specific survival (MSS) (*p* = 0.019, 0.017), and progression-free survival (PFS) (*p* = 0.043, 0.004, respectively). High intratumoral CD8+ and FoxP3+ lymphocyte count correlated with longer MSS (*p* = 0.036) and improved PFS (*p* = 0.047), respectively. Ulceration correlated with worse OS and worse MSS. Age, male gender, and higher stage (3 and 4) significantly correlated with worse survival. MCPyV positivity correlated with immune response. By multivariate analyses, only ulceration and age remained as independent predictors of worse OS; gender and stage remained for shorter PFS. Tumoral PD-L1 expression and increased density of intratumoral CD8+ lymphocytes and FoxP+ lymphocytes may represent favorable prognosticators in a subset of MCCs. Tumoral PD-L1 expression correlated with intratumoral CD8+ and FoxP3+ lymphocytes, which is supportive of an adaptive immune response.

## 1. Introduction

Merkel cell carcinoma (MCC) is a rare and aggressive primary cutaneous neuroendocrine carcinoma with frequent recurrences, metastases, high mortality rates, and rapidly increasing reported incidence [1]. Etiology for MCC includes ultraviolet (UV) radiation exposure, Merkel cell polyomavirus (MCPyV) infection, and immunosuppression [1]. Although MCPyV positivity and the presence of intratumoral CD8+ lymphocytes are favorable prognostic factors [2,3,4], there remains a need for additional biomarkers to stratify MCC risk.

Novel immunotherapies targeting the indoleamine 2,3-dioxygenase 1/tryptophan 2,3-dioxygenase/kynurenine/aryl hydrocarbon receptor (IDO1/TDO2-KYN-AhR) signaling cascade are currently under investigation. IDO1 is an intracellular cytoplasmic enzyme that catalyzes the degradation of tryptophan to kynurenine pathway metabolites [5]. The deregulation of tryptophan metabolism (decreasing tryptophan and increasing tryptophan metabolites) is connected with induction of the cell-cycle arrest and effector T-cell apoptosis and promotes Tregs activity, contributing to local immunosuppression [6]. Tumoral IDO1 expression can be variable in various cancers [7,8], and its expression in MCC has been reported in only one prior study where its high expression correlated with unfavorable clinical outcome [9].

Immunotherapies harness the immune system by targeting the programmed death receptor/ligand 1 (PD1/PD-L1) and accelerating adaptive immunologic response. By blocking the interaction between PD1 and PD-L1, inhibitors such as avelumab, nivolumab, and pembrolizumab have demonstrated significant improvements in clinical responses [10,11]. However, prognostic significance of PD-L1 expression in MCC remains still unclear [2,12,13,14]. Although the intratumoral CD8+ lymphocytes have been shown to be a favorable prognostic variable, the prognostic role FoxP3+ infiltrate in MCC has not been widely studied in MCCs.

In our study, we aim to analyze the following: (1) the immunohistochemical expression of PD-L1 and IDO1 in MCC cells and intratumoral CD8+ and FoxP3+ lymphocytes, and (2) the prognostic role of tumoral PD-L1 and IDO1 expression, and intratumoral CD8+ and FoxP3+ counts.

## 2. Results

One hundred and thirty-two patients were included in the study. The age of the patients ranged from 52 to 94 years (median, 77 years). Seventy-four were males and 58 were females. American Joint Committee on Cancer (AJCC) stages at time of diagnosis were I in 66, II in 53, III in 10, IV in 1, and unknown in 2 patients. Immunosuppression was seen in fifteen patients: chronic lymphocytic leukemia (*n* = 5), lymphoma (*n* = 4), renal transplant (*n* = 3), carcinoma (*n* = 2), and chronic hepatitis C (*n* = 1). The range of follow-up for all patients was 0 to 255 months (median, 22 months). Local recurrence and/or metastasis (progression) developed in 53/132 (40%) patients. Death was documented in 73/132 (55%) patients and 35/132 (26.5%) from MCC.

Eight-one (61%) patients received surgical treatment and 42 (32%) had sentinel lymph node biopsies. Fifty-one (39%) patients received adjuvant therapy in addition to surgical excision (18 with combined radiation and chemotherapy, 6 with only chemotherapy, and 27 with only radiation). Ten of these 51 patients received immunotherapy (two with nivolumab, three with pembrolizumab, four with avelumab, and one with both pembrolizumab and avelumab). The tumors of these 10 patients were positive for PD-L1 in 6 and negative in 4.

Sixty-three tumors (48%) were from the head and neck region and 69 (52%) were from other sites. The median tumor size and tumor thickness were 19 mm (range: 2 to 125 mm) and 10 mm (range: 1 to 55 mm), respectively. Mitoses were 1 to over 100 (median, 40). Ulceration, necrosis, perineural invasion, and lymphovascular invasion were present in 44 (33%), 43 (33%), 12 (9%), and 64 (48%) cases, respectively. Although most tumors exhibited immunoprofile characteristic for MCC, three tumors were CK20 negative and one rare tumor was positive for TTF1. Expression of MCPyV T-antigen was seen in 85/132 cases (64%).

**Figure 1:**
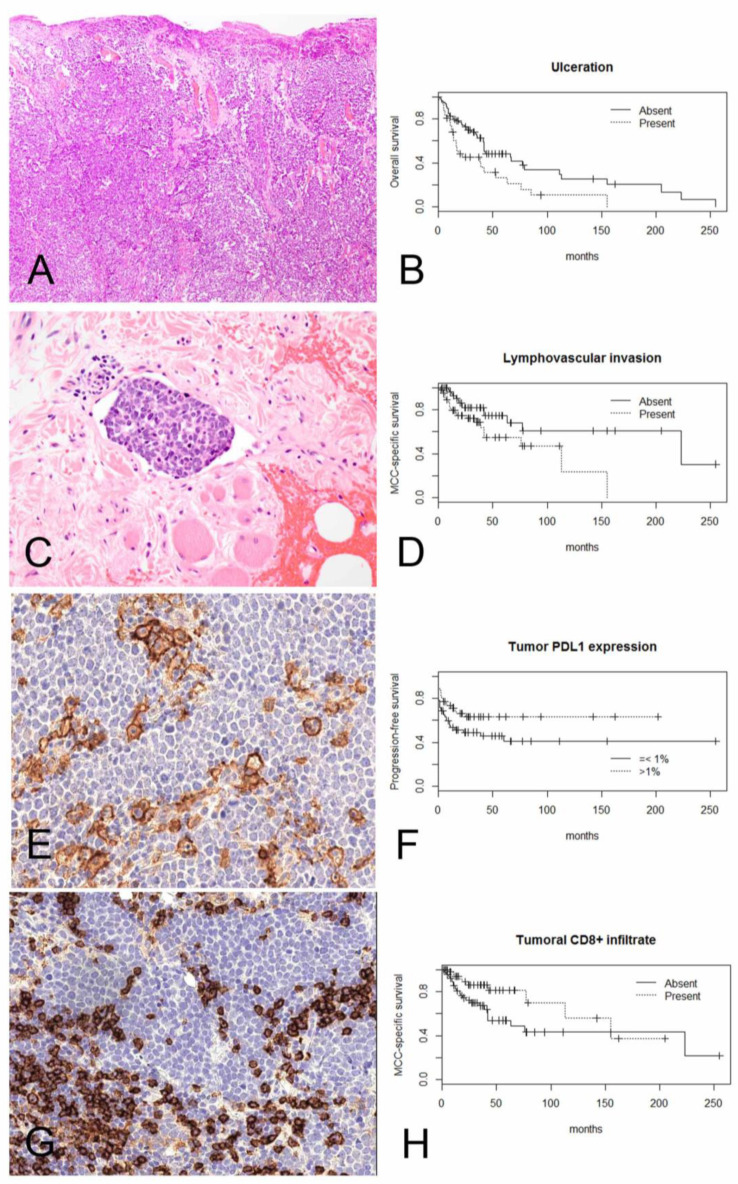
Kaplan–Meier curves demonstrate significant correlation between (**A**) presence of ulceration (10×) and (**B**) worse overall survival (log-rank *p* = 0.0039); (**C**) presence of lymphovascular invasion (40×) and (**D**) worse MCC-specific survival (log-rank *p* = 0.02); (**E**) membranous PD-L1 expression >1% of tumor cells (20×) and (**F**) improved progression-free survival (log-rank *p* = 0.041); (**G**) intratumoral CD8+ lymphocytes and (**H**) improved Merkel cell carcinoma-specific survival (log-rank *p* = 0.031).

Membranous tumoral PD-L1 expression >1% was seen in 48% (64/132) of cases (range, 0–70%). The median H-score of tumoral IDO1 expression was 155 (range, 0–210). There was co-expression of tumoral PD-L1 and IDO1 in 33 cases (33/130, 25%). The median counts of intratumoral CD8+ and FoxP3+ lymphocytes are 20.67 (range, 0–230) and 4.67 (range, 0–89.67), respectively. Cases with level of expression at median or above were considered high (Figure 1).

### 2.1. Positive MCPyV Status Correlated with Tumoral PD-L1 Expression and High Intratumoral CD8+ Cell Count

The clinicopathologic characteristics associated with tumoral PD-L1 expression, tumoral IDO1 expression, intratumoral CD8+ cell count, and intratumoral FoxP3+ cell count evaluated with Fisher’s exact tests are summarized in Table 1. Tumoral PD-L1 expression >1% significantly correlated with tumor thickness >10 mm (*p* = 0.0048) and positive MCPyV status (*p* = 0.018). High intratumoral CD8+ cell count significantly correlated with absence of ulceration (*p* = 0.0092) and positive MCPyV status (*p* = 0.018).

### 2.2. Tumoral PD-L1 Expression Correlated with Intratumoral CD8+ and FoxP3+ Lymphocytes, Which Is Supportive of an Adaptive Immune Response

By linear regression analyses, correlations between tumoral PD-L1 expression with intratumoral CD8+ cell count (*p* < 0.0001) and intratumoral FoxP3+ cell count (*p* < 0.0001), and intratumoral CD8+ cell count with intratumoral FoxP3+ cell count (*p* < 0.0001) were observed. There was no correlation between tumoral IDO1 expression and MCPyV status versus tumoral PD-L1 expression, intratumoral CD8+, and FoxP3+ cell counts.

### 2.3. Tumoral PD-L1 Expression and Increased Density of Intratumoral CD8+ Lymphocytes and FoxP3+ Lymphocytes May Positively Impact Survival in a Subset of MCCs

**Figure 2:**
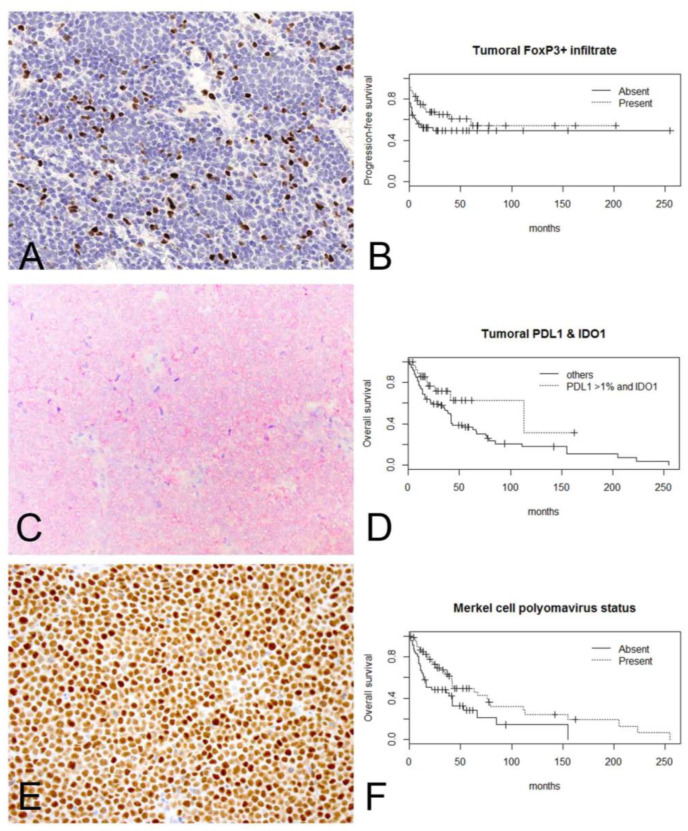
Kaplan–Meier curves demonstrate significant correlation between (**A**) intratumoral FoxP3+ lymphocytes (20×) and (**B**) improved progression-free survival (log-rank *p* = 0.044); (**C**) combined tumoral PD-L1 >1% and high IDO1 expression (40×) and (**D**) improved overall survival (log-rank *p* = 0.052); (**E**) nuclear expression of Merkel cell polyomavirus large T-antigen (40×) and (**F**) improved overall survival (log-rank *p* = 0.0066).

**Figure 3:**
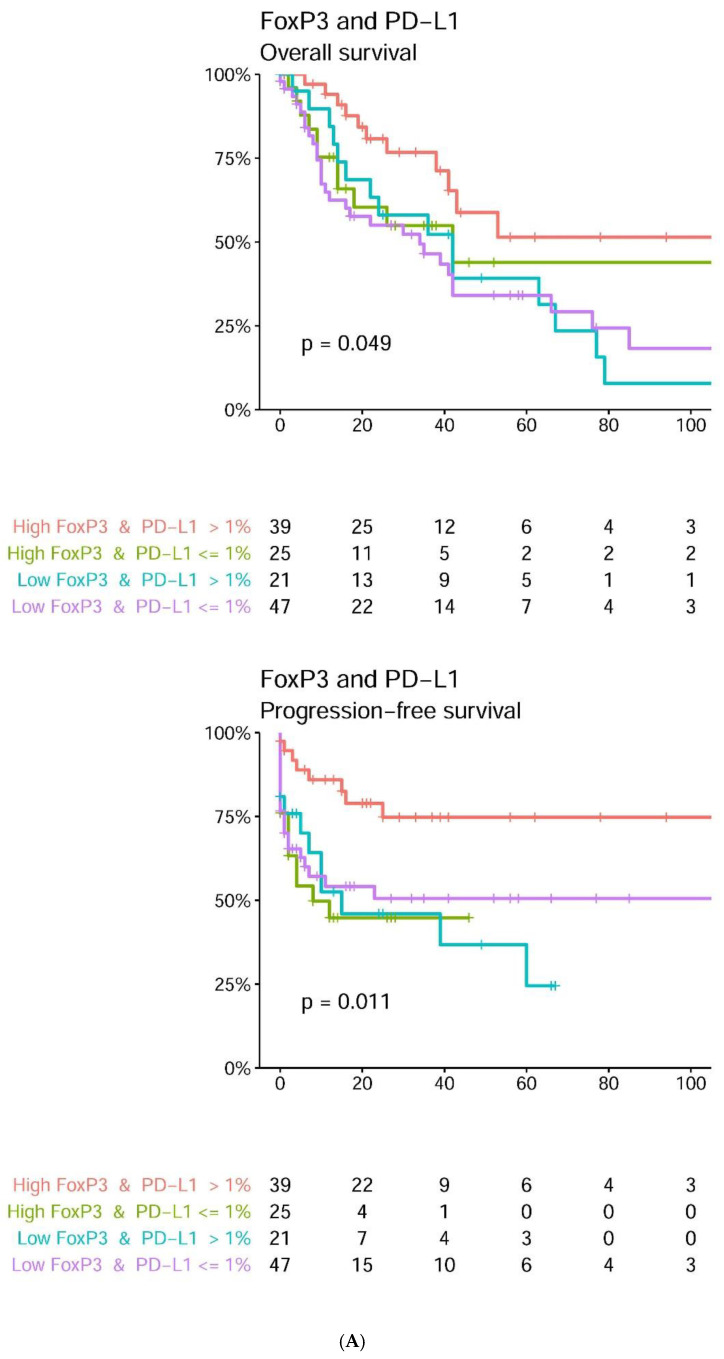

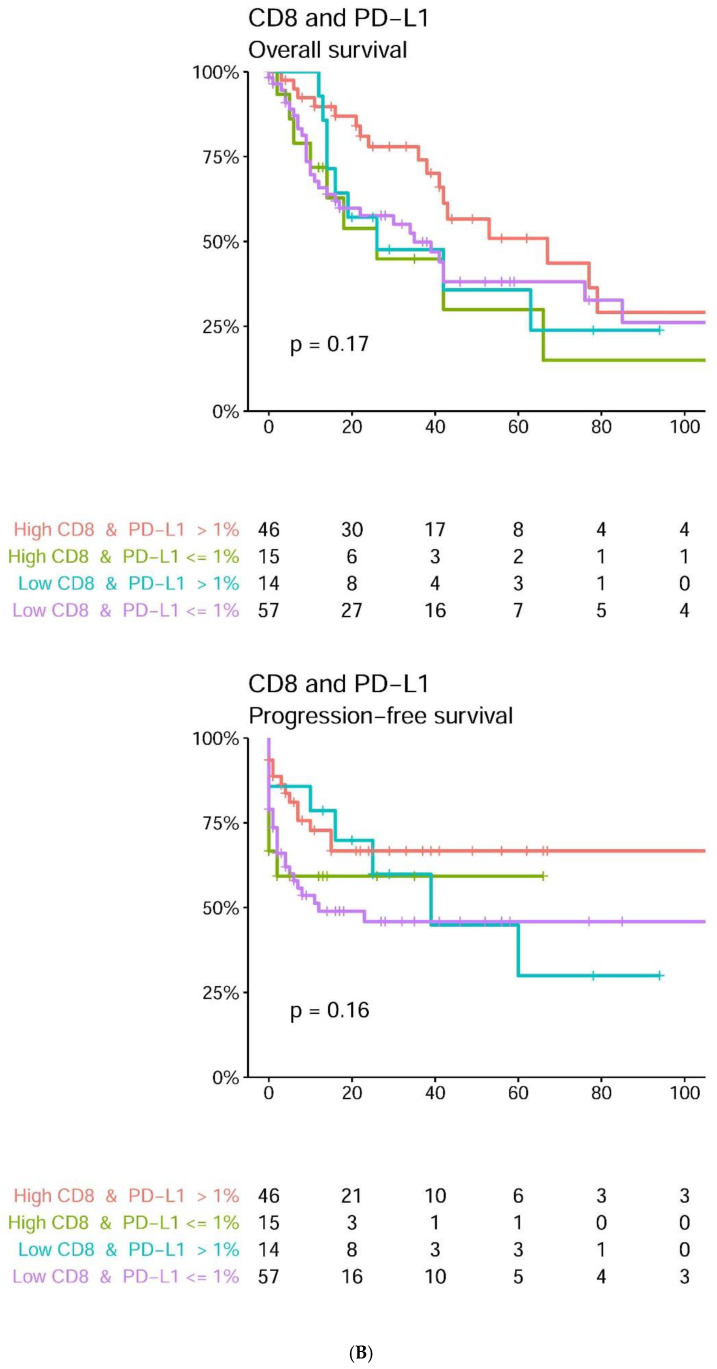

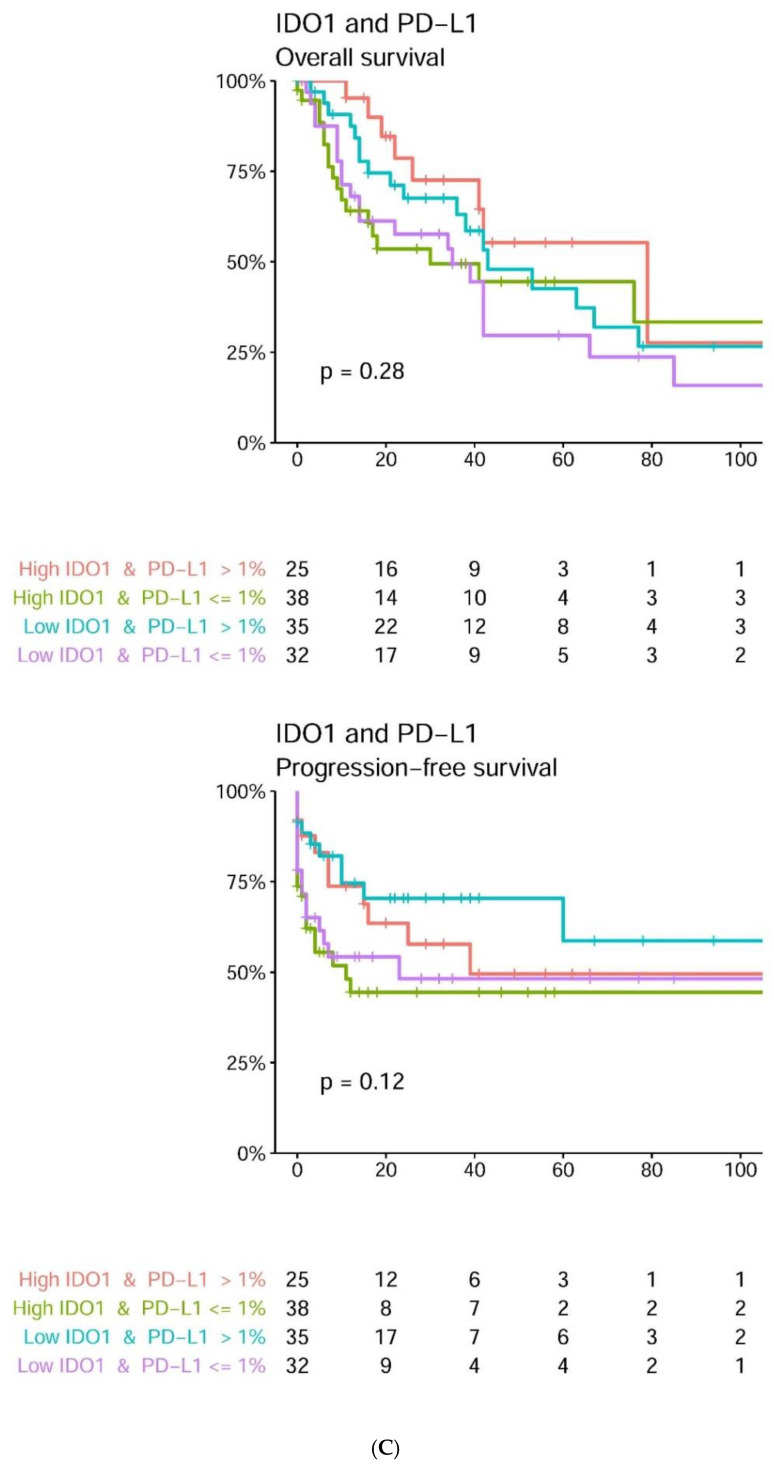
(**A**) Kaplan–Meier curves illustrate the associations between tumoral PD-L1 expression and intratumoral FoxP3+ cell count for overall survival and progression-free survival. (**B**) Kaplan–Meier curves illustrate the associations between tumoral PD-L1 expression and intratumoral CD8+ cell count for overall survival and progression-free survival. (**C**) Kaplan–Meier curves illustrate the associations between tumoral PD-L1 expression and tumoral IDO1 expression for overall survival and progression-free survival.

By univariate analyses, tumoral PD-L1 expression >1% and combined tumoral PD-L1 >1% and high intratumoral FoxP3+ cell count correlated with improved overall survival (OS) (*p* = 0.016, 0.0072), MCC-specific survival (MSS) (*p* = 0.019, 0.017), and progression-free survival (PFS) (*p* = 0.043, 0.004, respectively) (Table 2, Figure 2 and Figure 3). High intratumoral CD8+ and FoxP3+ cell counts correlated with longer MSS (*p* = 0.036) and improved PFS (*p* = 0.047), respectively. Combined tumoral PD-L1 >1% and high tumoral IDO1 expression correlated with improved OS (Figure 2), but this result only approached statistical significance (*p* = 0.057) (Table 2). Expression of MCPyV T-antigen correlated with improved overall survival (OS) (*p* = 0.0076) and MSS (*p* = 0.04). Age (*p* = 0.00067) significantly correlated with worse OS. Male gender and higher stage (3 and 4) significantly correlated with worse MSS (*p* = 0.029 and 0.038, respectively) and shorter PFS (*p* = 0.006 and < 0.0001, respectively). Ulceration correlated with worse OS (*p* = 0.0046) and worse MSS (*p* = 0.008). Lymphovascular invasion was significantly associated with shorter MSS (*p* = 0.02) (Table 2).

### 2.4. By Multivariate Analyses, Ulceration and Age Are Independent Predictors of Worse OS; and Gender and Stage Are Independent Predictors of Shorter PFS

Covariates being significant in univariate analyses (ulceration, lymphovascular invasion, MCPyV large T-antigen, tumoral PD-L1 >1%, tumoral PD-L1 >1% and high intratumoral FoxP3+ cell count, tumoral PD-L1 >1% and high intratumoral CD8 cell count, intratumoral CD8+ and FoxP3+ cell counts, intratumoral CD8+ cell count, intratumoral FoxP3+ cell count, age, gender, site, and stage) were included in the Cox multivariate model. By multivariate analyses, only ulceration (*p* = 0.03) and age (*p* = 0.0031) remained as independent predictors of worse OS (*p* = 0.046); and gender (*p* = 0.018) and stage (*p* < 0.0001) remained as independent predictors for shorter PFS (Table 3).

## 3. Discussion

In the current study tumoral PD-L1 expression >1% significantly correlated with better OS, MSS, and PFS in univariate analysis. Similarly, Lipson et al. [12] reported expression of tumoral PD-L1 to be independently associated with better OS in MCC patients. A possible mechanism of enhanced tumoral PD-L1 expression and better clinical outcome could be connected with the hypomethylation of PD-L1 gene promoter. Findings reported by Micevic et al. [15] suggest that the hypomethylation of PD-L1 (and possible subsequent increased protein expression of PD-L1) is associated with a phenotype of immune activation and expression of interferon signaling pathway genes in cutaneous melanoma. This hypothesis needs further investigations in MCC patients, because some studies did not confirm the significant prognostic impact of PD-L1 on survival in MCC patients [2,13,14].

In our cohort, tumoral IDO1 expression had no prognostic impact on survival in univariate analysis. In the majority of previous studies tumoral IDO1 expression was correlated with a less favorable clinical outcome [7], but others reported a positive prognostic effect on long-term survival [8]. There are two hypotheses that might explain this putative contradiction. First, local tryptophan depletion has been reported to decrease the proliferation of tumor cell lines in an in vitro model [16,17]. Second, the IFN-γ is a crucial cytokine to induce IDO1 expression [17]. During an antitumor immune response, IFN-γ and other pro-inflammatory cytokines are secreted, and IDO1 expression could be a marker of an ongoing antitumoral immune response.

Interestingly, in the current study, there was no correlation between tumoral IDO1 expression and intratumoral CD8+ and FoxP3+ cell counts, supporting a different mechanism of induction of IDO1 expression within the tumor microenvironment of MCC [18,19]. Wardhani et al. [9] reported that higher tumoral IDO1 expression was significantly correlated with worse OS in 43 MCC patients, and enhanced tumoral IDO1 immunoreactivity was correlated with MCPyV-positive status. We did not observe any significant correlation between tumoral IDO1 expression with MCPyV status and clinicopathologic variables.

The association between tumoral IDO1 expression and the efficacy of IDO1 inhibitor is unknown at the current time. However, the co-expression of tumoral PD-L1 and IDO1 raises a possibility regarding therapeutic role of combined IDO1 inhibitor and PD1/PD-L1 inhibitor for MCC. Clinical trials of epacadostat, an IDO1 inhibitor, as single agent for MCC (UCDCC#271) and in combination with pembrolizumab/nivolumab for patients with advanced solid tumors are currently under investigation [20].

Forkhead transcription factor FoxP3 is a master regulator of Treg development. Although FoxP3+ infiltrate in MCC was previously studied by several studies, its prognostic value has not been widely studied in MCCs [21,22,23]. High intratumoral FoxP3+ infiltrate correlated with improved survival in our study. In accordance with our results, Sihto et al. [3] revealed that a high number of intratumoral FoxP3+ lymphocytes was associated with favorable outcome in MCC patients. Interestingly, concomitant intratumoral PD-L1 expression and high intratumoral FoxP3+ lymphocytes exhibited better OS, MSS, and PFS in univariate analyses in our study. The association between PD-L1 and FoxP3 that has been reported with PD-L1 influences the conversion of naïve T cells to Tregs and upregulates FoxP3 expression [24].

A strong association of tumoral PD-L1 expression and high intratumoral CD8+ lymphocytes was observed in our study; however, concomitant tumoral PD-L1 expression and high intratumoral CD8 infiltrate did not correlate with survival. Similar to observations made by Lipson et al. [12] and Mitteldorf et al. [25], tumoral PD-L1 expression in our cases appear to localize to areas with tumor-infiltrating lymphocytes. These results are in line with findings of previous study in which tumoral PD-L1 expression was correlated with CD8+ lymphocyte infiltration [26,27]. These findings suggest that tumoral PD-L1 expression could be a result of an adaptive immune response against viral and/or neoplastic antigens.

Several previous studies suggest that intratumoral CD8+ lymphocytes are strongly correlated with better survival in MCC [2,3,4,28]. The findings of our study confirmed that high intratumoral CD8+ cell count had a positive prognostic impact on survival in MCC patients and highlighted the crucial role of cytotoxic CD8+ lymphocytes in controlling disease progression.

In our cohort, MCPyV T-antigen expression significantly correlated with improved OS, and its expression had a strong association with tumoral PD-L1 expression and high intratumoral CD8+ cell count. In line with our results, Harms et al. [29] revealed that MCPyV-positive tumors were associated with significantly increased CD8+ cells in comparison to MCPyV-negative tumors. Lipson et al. [12] reported a significant association of the presence of MCPyV with tumor PD-L1 expression. Sihto et al. [3] showed significant correlation between MCPyV DNA-positive MCCs and higher numbers of CD8+ and FoxP3+ lymphocytes as compared with MCPyV-negative tumors. In contrast, Feldemeyer et al. [13] observed no significant differences between MCPyV status and PD-L1 expression in tumoral cells.

We observed tumoral PD-L1 expression in MCPyV-positive tumors more frequently than MCPyV-negative MCCs (48 versus 16, *p* = 0.018) in our series, supporting the hypothesis that virus-positive and virus-negative MCCs have different immune microenvironment. These findings suggest that MCPyV-related immune response upregulates tumoral PD-L1 and tumoral IDO1 expression and intratumoral CD8+ infiltrate.

Although tumoral PD-L1 expression and concomitant tumoral PD-L1 expression and high intratumoral FoxP3+ infiltrate correlated with improved survival in univariate analyses; the presence of ulceration, age, gender, and stage remain as independent predictors of survival.

## 4. Materials and Methods

One hundred and thirty two patients with a diagnosis of primary cutaneous MCC rendered between 1995 and 2018 were retrieved from the pathology archives of six clinical institutions in Poland, Taiwan, and the United States. The diagnoses were histopathologically confirmed by the contributing pathologists (PD, C-LW, JK and JR) and the corresponding author (MPH). Medical data including clinical notes, imaging results, and histopathology reports were reviewed to confirm that the analyzed tumor is a primary cutaneous MCC and not a metastasis of visceral-located neuroendocrine carcinoma. The study was approved by Institutional Review Boards.

### 4.1. Clinical Findings and Histologic Features

All tumors have diagnostic features of MCC: compatible histologic appearance, expression of keratin 20 and neuroendocrine markers (chromogranin, synaptophysin, neuron specific enolase, and/or CD56). Neurofilament, EMA, keratin 7, or other keratins (AE1/AE3, CAM5.2, pan or wide spectrum keratin) were performed in a subset of cases. Thyroid transcription factor 1 (TTF1) was performed in the majority of cases. Detailed histopathologic parameters (tumor size, growth pattern (nodular, infiltrative, or mixed), ulceration, tumor thickness, mitotic rate (per squared millimeters), lymphovascular invasion (LVI), perineural invasion (PNI), necrosis, and involvement of the overlying epidermis (epidermotropism) were assessed by the contributing pathologists (PD, C-LW, JK, JR) and confirmed by the corresponding author (MPH). The utilized tumor size cut-offs were those established by the 8th edition American Joint Committee on Cancer (AJCC) staging system for MCC. The following data were extracted from medical records: age of the patients, gender, lesion site, date of biopsy, disease status over time, and at last follow-up (recurrence, metastasis).

### 4.2. Immunohistochemistry

Tissue microarrays composed of two 2 mm-tissue cores from each tumor were constructed. Immunohistochemical studies were performed on 5-micrometer-thick tissue sections using a Bond 3 automated immunostainer (Leica Microsystems, Bannockburn, IL, USA), with primary antibodies against CD8 (4B11, undiluted, Leica Microsystems), FoxP3 (236A/E7, undiluted, BioCare Medical, Concord, CA, USA), IDO1 (1F8.2, 1:400, Millipore, Burlington, MA, USA), MCPyV large T-antigen (CM2B4, sc-136172, 1:100, Santa Cruz Biotechnology, Santa Cruz, CA, USA), and PD-L1 (E1L3N, 1:200, Cell Signaling Technology, Danvers, MA, USA).

Scoring of the cases was done blinded to clinical outcomes by two independent authors (PD and MPH). The percentage of tumor cells with positive PD-L1 membranous staining of any intensity was evaluated with greater than 1% considered positive. The cytoplasmic IDO1 and nuclear CM2B4 staining of tumor cells was done using the H-score [(percentage at 1+) × 1 + (percentage at 2+) × 2 + (percentage at 3+) × 3]. Any CM2B4 expression was considered positive. Total cell counts of intratumoral CD8+ and FoxP3+ lymphocytes were evaluated in three consecutive images captured at 40× magnification of the hotspot. The median number of positive cells was used as a cut-off value for high and low CD8+ or FoxP+ cases.

### 4.3. Statistical Analysis

The statistical association between tumoral expression of PD-L1 and IDO1, intratumoral CD8+ and FoxP3+ cell count, and clinicopathologic features (tumor size, tumor thickness, growth pattern, mitotic index, ulceration, necrosis, lymphovascular invasion, and perineural invasion) were evaluated by Fisher’s exact tests. The *p*-values were corrected for false discovery rate for 4 potential biomarkers. MCC-specific survival (MSS) and overall survival (OS) were defined as the number of months from initial diagnosis of MCC to patient’s death related to MCC and by any cause, respectively. Progression-free survival (PFS) was defined as the number of months from initial diagnosis to identification of local recurrence or metastases in lymph nodes and/or distant organs. Kaplan–Meier curves and log-rank tests were performed to assess the differences in MSS, OS, and PFS between subgroups. The Cox proportional hazards model was performed. All statistically significant parameters from univariate Cox models were included in multivariate analysis. The proportionality assumptions of the Cox models were tested. Linear regression was used for plotting tumoral PD-L1 percent expression against tumoral IDO1 H-score, intratumoral CD8+ and intratumoral FoxP3+ cell count; intratumoral CD8+ against intratumoral FoxP3+ cell count and tumoral IDO1 H-score; and CM2B4 H-score against percent tumoral PD-L1 percent expression, tumoral IDO1 H-score, and intratumoral CD8+ and intratumoral FoxP3+ cell count. All analyses and plots were performed using the R statistical package [30]. A two-tailed *p*-value of less than 0.05 was considered statistically significant.

## 5. Conclusions

Tumoral PDL1 expression and increased density of intratumoral CD8+ lymphocytes and FoxP3+ lymphocytes may positively impact on survival in a subset of MCCs. Tumoral PDL1 expression correlated with intratumoral CD8+ and FoxP3+ lymphocytes, which is supportive of an adaptive immune response. There is a correlation between immune response and MCPyV positivity.

## Figures and Tables

**Table 1:** Summary of clinical, histologic, and immunohistochemical characteristics.

	PD-L1 *n* = 132			CD8 *n* = 132			FoxP3 *n* = 132			IDO1 *n* = 130		
	High	Low	*p*-Value	High	Low	*p*-Value	High	Low	*p*-Value	High	Low	*p*-Value
Age												
>77 years	27	38	0.48	32	32	0.65	29	36	0.86	33	30	0.65
<=77 years	37	30		29	39		31	36		30	37	
Gender												
Male	37	36	0.72	31	42	0.72	29	44	0.64	34	39	0.72
Female	27	32		30	29		31	28		29	28	
Site												
Head and neck	32	30	0.73	30	32	0.73	33	31	0.56	27	35	0.56
Other sites	32	38		31	39		27	41		36	32	
Size												
>20 mm	36	23	0.07	27	32	1	26	34	1	32	27	0.48
<=20 mm	28	45		34	39		33	39		31	40	
Thickness												
>10 mm	39	23	0.019 *	30	34	1	26	37	0.64	34	29	0.58
<=10 mm	25	43		30	36		32	35		28	37	
Ulceration												
Present	20	24	0.71	13	31	0.037 *	15	29	0.14	24	20	0.48
Absent	44	44		48	40		45	43		39	47	
Mitoses												
>40/mm^2^	31	33	1	25	40	0.32	25	40	0.32	30	34	1
<=40/mm^2^	33	34		35	31		34	32		32	33	
Growth pattern												
Nodular	34	26	0.44	26	34	0.73	31	29	0.44	28	32	0.73
Nodular and infiltrative, infiltrative	30	42		35	37		29	43		35	35	
Necrosis												
Present	22	21	0.71	17	27	0.48	17	26	0.48	25	18	0.48
Absent	42	47		44	44		43	46		38	49	
Lymphovascular invasion												
Present	33	31	0.60	28	36	0.60	25	39	0.34	36	26	0.21
Absent	31	37		33	35		35	33		27	41	
Perineural invasion												
Present	6	6	1	4	8	1	4	8	1	6	6	1
Absent	58	62		57	63		56	64		57	61	
MCPyV												
Present	48	37	0.036 *	46	39	0.036 *	42	43	0.27	45	38	0.15
Absent	16	31		15	32		18	29		18	29	

* *p* < 0.05, statistically significant; *p* < 0.09, approaching statistical significance. *p*-values are after correction for multiple hypothesis testing (False Discovery Rate in each row).

**Table 2:** Univariate Cox proportional hazards model.

		Overall Survival		Merkel Cell Carcinoma-Specific Survival		Progression-Free Survival	
	N	Hazard Ratio	*p*-Value	Hazard Ratio	*p*-Value	Hazard Ratio	*p*-Value
PD-L1 >1%	132	0.55	0.016 *	0.41	0.019 *	0.56	0.043 *
CD8-high	132	0.76	0.25	0.45	0.036 *	0.62	0.098
FoxP3-high	132	0.66	0.092	0.62	0.17	0.57	0.047 *
IDO1-high	130	0.93	0.75	1.15	0.69	1.29	0.36
CD8-high FoxP3-high	132	0.66	0.13	0.41	0.046 *	0.58	0.091
PD-L1 >1% FoxP3-high	132	0.43	0.0072 *	0.28	0.017 *	0.35	0.004 *
PD-L1 >1% CD8-high	132	0.57	0.053	0.28	0.018 *	0.45	0.024 *
PD-L1 >1% IDO1-high	130	0.50	0.057	0.87	0.74	1.14	0.66
Size (>20 mm)	132	1.03	0.91	1.31	0.44	1.20	0.50
Thickness (>10 mm)	132	1.04	0.89	1.14	0.71	1.06	0.84
Growth pattern (nodular versus infiltrative/mixed)	132	0.69	0.12	0.58	0.12	0.57	0.051
Ulceration	132	2.01	0.0046 *	2.51	0.008 *	1.67	0.067
Mitoses (>40/mm^2^)	132	0.97	0.89	1.28	0.47	1.17	0.58
Necrosis	132	1.30	0.32	1.19	0.66	1.45	0.19
Lymphovascular invasion	132	1.50	0.095	2.29	0.02 *	1.51	0.14
MCPyV large T-antigen	132	0.52	0.0076 *	0.49	0.04 *	0.71	0.22
Age (>versus ≤77 years)	132	2.35	0.00067 *	1.33	0.41	0.9	0.7
Gender (male versus female)	132	1.29	0.28	2.23	0.029 *	2.29	0.006 *
Site (other versus head & neck)	132	1.32	0.26	0.76	0.44	0.48	0.012 *
Stage (3–4 versus 1–2)	132	1.27	0.62	2.75	0.038 *	27.2	<0.0001 *
Immunosuppression	132	1.18	0.63	1.2	0.71	1.02	0.97
Treatment (adjuvant therapy versus surgery	132	1.13	0.63	1.82	0.094	1.52	0.13

MCPyV: Merkel cell polyomavirus. * *p* < 0.05, statistically significant; *p* < 0.09, approaching statistical significance.

**Table 3:** Multivariate Cox proportional hazards model.

		Overall Survival		Merkel Cell Carcinoma-Specific Survival		Progression-Free Survival	
	N	Hazard Ratio	*p*-Value	Hazard Ratio	*p*-Value	Hazard Ratio	*p*-Value
PD-L1 >1%	132	0.92	0.78	0.84	0.76	1.48	0.37
Ulceration	132	1.79	0.03 *	1.73	0.18	-	-
MCPyV T-antigen expression	132	0.73	0.24	0.72	0.41	-	-
PD-L1 >1% FoxP3-high	132	0.54	0.13	0.99	0.99	0.32	0.058
PD-L1 >1% CD8-high	132	-	-	0.27	0.15	0.70	0.48
CD8-high FoxP3-high	132	-	-	0.44	0.26	-	-
Lymphovascular invasion	132	-	-	1.94	0.1	-	-
CD8	132	-	-	2.4	0.24	-	-
FoxP3	132	-	-	-	-	1.35	0.41
Age	132	2.13	0.0031 *	-	-	-	-
Gender	132	-	-	1.78	0.19	2.08	0.018 *
Site (other versus head & neck)	132	-	-	-	-	0.56	0.064
Stage (3–4 versus 1–2)	132	-	-	2.37	0.12	20.57	<0.0001 *

MCPyV: Merkel cell polyomavirus. * *p* < 0.05, statistically significant; *p* < 0.09, approaching statistical significance.

## Data Availability

The data presented in this study are available in this article.
